# The Effects of Attractiveness and Status on Personality Evaluation

**DOI:** 10.5964/ejop.v11i4.896

**Published:** 2015-11-27

**Authors:** Stefano Tartaglia, Chiara Rollero

**Affiliations:** a Department of Psychology, University of Turin, Turin, Italy; b Faculty of Psychology, University eCampus, Novedrate, Italy; Academy of Special Education, Warsaw, Poland

**Keywords:** gender, personality evaluation, stereotyping, attractiveness, status, experimental design

## Abstract

Research on personality has shown that perceiving a person as attractive fosters positive expectations about his/her personal characteristics. Literature has also demonstrated a significant link between personality traits and occupational achievement. Present research examines the combined effects of attractiveness, occupational status, and gender on the evaluation of others’ personality, according to the Big Five model. The study consisted of a 2 (Attractiveness: High vs. Low) x 2 (occupational Status: High vs. Low) x 2 (Target gender: Male vs. Female) between-subjects experimental design (N = 476). Results showed that attractive targets were considered more positively than unattractive targets, and this effect was even stronger for male targets. Occupational status influenced perceived agreeableness (lower for high-status targets) and perceived conscientiousness (higher for high-status targets).

According to differential psychologists, personality can be effectively and parsimoniously described by models composed of three to seven major traits (Revelle, Condon, & Wilt, 2011; Swami et al., 2013). Among these approaches, the most widely-accepted is the five-factor model of personality, which refers to the 'Big Five' dimensions of Openness to Experience, Conscientiousness, Extraversion, Agreeableness, and Neuroticism (Digman, 1990; Goldberg, 1993). This model explains individual differences in personality at a broad level of abstraction (Costa & McCrae, 1992; Goldberg, 1993; McCrae & Costa, 1999) and shows good cross-cultural applicability (McCrae & Costa, 1997): recurring regularities – despite differences in history, religion, language and culture – suggest that these personality traits are basic characteristics of the human species. The Big Five dimensions proved to be universal both for self-evaluation and from the observer’s perspective when used to evaluate personality of other people (McCrae & Terracciano, 2005).

Considering gender differences, Costa, Terracciano, and McCrae (2001) reported pancultural patterns of differences between men and women. Specifically, women are higher than men in Extraversion (Costa et al., 2001; McCrae & Terracciano, 2005), but not in the work setting (Robinson, 2009). Women also score higher than men on Conscientiousness (Feingold 1994; McCrae & Terracciano, 2005) and Neuroticism (Costa et al., 2001; McCrae & Terracciano, 2005). Several studies reported also higher level of Agreeableness in women (Costa et al., 2001), with very few exceptions across countries (i.e. in Italy, where no gender differences emerged, McCrae & Terracciano, 2005).

Research on personality has been developed in several directions. One of these is the study of stereotypic processes fostering inferences on others’ personality based on cognitive biases (i.e. Ćurković & Franc, 2010; Langlois et al., 2000). In this perspective, studies aim at investigating how individuals form impressions of others and how specific characteristics influence the expectations. The present study follows this perspective focusing on two characteristics: attractiveness and status. The first one is a classical basis of stereotyping. The second one has been studied mainly as an outcome of personality traits (i.e. Dilchert & Ones, 2008; Judge, Higgins, Thoresen, & Barrick, 1999), whereas here it is considered as a predictor of different personality inferences.

## Inferences on Personality: What is Beautiful is Good

The ‘‘what is beautiful is good’’ stereotype (Dion, Berscheid, & Walster, 1972) is a classic phenomenon in social psychology and implies that when forming impressions of others, perceiving a person as good looking fosters positive expectations about personal characteristics. Physical attractiveness has been shown to generate a broadly favorable impression of both men and women (Langlois et al., 2000). Indeed, people seem to assume that positive interpersonal qualities and physical attractiveness are systematically linked (i.e., a “halo effect”) (Andreoni & Petrie, 2008; Callan, Powell, & Ellard, 2007; Smith, McIntosh, & Bazzini, 1999). The effects of attractiveness are strong and pervasive. As Langlois et al. (2000) underline in their meta-analysis, attractiveness is a noteworthy advantage for both children and adults in almost every domain. Based on the “what is beautiful is good” effect (Dion et al., 1972), several studies (Eagly, Ashmore, Makhijani, & Longo, 1991; Feingold, 1992; Langlois et al., 2000) demonstrated that this phenomenon functions as a stereotype, making the perceived link between appearance and personality larger than the actual link. Attractive targets are assessed as less introverted, neurotic and higher on Openness dimension than unattractive targets (Ćurković & Franc, 2010; Langlois et al., 2000). In sum, attractiveness leads perceivers to make strong inferences of personality goodness.

## When Attractiveness and Status Can Be Detrimental: The Beauty is Beastly Effect

Despite the above-mentioned studies attesting the positive effects of physical attractiveness when forming impressions, a small body of research suggests that attractiveness can be detrimental to women in certain situations. The “beauty is beastly” effect (Heilman & Saruwatari, 1979; Heilman & Stopeck, 1985) posits that attractive women are at an advantage for feminine sex-typed jobs and at a disadvantage for masculine sex-typed jobs. Although the literature concerning the “beauty is beastly” effect is less consistent than studies on the “what is beautiful is good” effect, recently Johnson, Podratz, Dipboye, and Gibbons (2010) confirmed its validity in definite situations. Specifically, attractiveness can be detrimental for attractive women applying for masculine jobs, mainly for high-status positions, when physical appearance is perceived to be unimportant (Rollero & Tartaglia, 2013). Thus, attractiveness and its relation with professional status are of various significance depending on the gender of the person being judged.

## Personality and Status

Many researchers interested in the consequences of personality traits have considered the Big Five dimensions as predictive elements of status attainment in the occupational domain (i.e. Judge et al., 1999). Literature has largely demonstrated that employees with a certain personality trait profile work harder and earn a higher salary (Barrick & Mount, 1991; Dilchert & Ones, 2008; Ng, Eby, Sorensen, & Feldman, 2005; Seibert & Kraimer, 2001). Specifically, Conscientiousness and Extraversion show a positive relationship with salary and job satisfaction (Judge, Heller, & Mount, 2002; Sutin, Costa, Miech, & Eaton, 2009) whereas Neuroticism and Agreeableness correlate negatively with success criteria, occupational level, and job satisfaction (Cohrs, Abele, & Dette, 2006; Rode, Arthaud-Day, Mooney, Near, & Baldwin, 2008). Lastly, the results obtained in studies on Openness have been inconsistent (Furnham, Taylor, & Chamorro-Premuzic, 2008; Ng et al., 2005). Thus, if literature underlines that personality paves the way to professional status, the actual relation between status and personality may be the basis for stereotyping people on the ground of their social position. In this sense, studies about the relation between status and personality may be a good starting point to assess the opposite direction: people may infer others’ characteristics on the basis of their professional status.

## The Current Study

The aim of this study was to investigate the effects of relevant stereotypes in the context of the Big Five model. As seen, literature has explored the relation between personality and occupational status, as well as the influence of attractiveness stereotype on the evaluation of others. However, to our knowledge no study has examined the combined effects of attractiveness, occupational status, and gender on the evaluation of others’ personality. The present study examined whether attractiveness, occupational status and gender of the target affect people’s evaluations in relation to five personality traits: Extraversion, Agreeableness, Neuroticism, Conscientiousness, and Openness to Experience. The study consisted of a 2 (Attractiveness: High vs. Low) x 2 (target occupational Status: High vs. Low) x 2 (Target gender: Male vs. Female) between-subjects experimental design.

## Hypotheses

On the ground of research literature, we set the following hypotheses.

Hypothesis 1. Both targets’ attractiveness and occupational status should increase perceived Extraversion. The strongest available conclusions come from the meta-analysis of Langlois et al. (2000) who found that observer inferences of attractiveness were correlated with observer reports of Extraversion. Conversely, unattractive targets are assessed as more introverted (Ćurković & Franc, 2010). Concerning status, positive relationships have been found between Extraversion and job success criteria (Boudreau et al., 2001; Judge et al., 1999; Rode et al., 2008). Because the status manipulation refers to the work context, following Robinson (2009) we did not expect any effect of the target gender on perceived Extraversion.

Hypothesis 2. Previous research (Ćurković & Franc, 2010; Langlois et al., 2000) did not show any effect of attractiveness on Agreeableness. Instead, high occupational status should decrease the evaluation of Agreeableness of the target, as most studies have found a negative relationship between Agreeableness and occupational status (Boudreau et al., 2001; Judge et al., 1999; Ng et al., 2005; Rode et al., 2008; Seibert & Kraimer, 2001). Although several studies reported higher levels of Agreeableness in women (Costa et al., 2001), a cross-cultural study conducted in 50 different cultures did not find that effect in the Italian sample (McCrae & Terracciano, 2005). For this reason, we did not hypothesize any influence of target gender on perception of Agreeableness.

Hypothesis 3. Target’s attractiveness, occupational status and gender were expected to affect perceived Neuroticism. On the basis of the ‘beauty is good’ effect (Dion et al., 1972), physically attractive individuals are perceived as having various positive qualities (e.g., Eagly et al., 1991; Feingold, 1992; Langlois et al., 2000), whereas unattractive targets are assessed as more emotionally unstable (Ćurković & Franc, 2010). Concerning status, several studies have found that Neuroticism correlates negatively with job success criteria (Boudreau et al., 2001; Judge et al., 1999; Ng et al., 2005; Rode et al., 2008) Finally, as many studies report higher levels of Neuroticism among women (Costa et al., 2001; McCrae & Terracciano, 2005), we expected female target to be evaluated as more neurotic.

Hypothesis 4. Target’s attractiveness, occupational status and gender were expected to influence the perception of Conscientiousness. On the ground of the ‘beauty is good’ effect (Dion et al., 1972), more attractive individuals should be judged as more conscientious. Moreover, most results indicate a positive relationship between this trait and job status (Barrick & Mount, 1991; Judge et al., 2002; Judge et al., 1999; Sutin et al., 2009). Finally, we expected that female targets should be considered more conscientious because many studies report higher level of Conscientiousness in women in respect to men (Feingold 1994; McCrae & Terracciano, 2005).

Hypothesis 5. Attractiveness should increase perceived Openness, as unattractive targets are assessed as lower on this trait (Ćurković & Franc, 2010). We did not hypothesize other influences because the results on the relation of occupational status and target gender with Openness have been inconsistent. Positive (Ng et al., 2005) or negative (Furnham et al., 2008) associations between Openness and occupational status have been found, whereas other studies showed no association at all (Barrick & Mount, 1991; Boudreau et al., 2001; Judge et al., 1999). Concerning Target gender, in a cross cultural study McCrae and Terracciano (2005) found that different traits related to Openness personality factor have different relations with target gender (i.e. men are higher in Openness to ideas whereas women in Openness to feelings).

## Method

### Participants and Experimental Design

A sample of 476 university students (48.2% male and 51.8% female) participated in the present study. Participants were recruited among undergraduate and graduate students of Arts and Science Schools in Italy. Their average age was 23.22 years (*SD* = 3.35). The ethnic composition of the sample was completely homogeneous: all participants were Italians. The study consisted of a 2 (Attractiveness: High vs. Low) x 2 (Occupational Status: High vs. Low) x 2 (Target Gender: Male vs. Female) between-subjects experimental design.

### Procedure

A photograph (2.95 in. [7.5 cm] x 2.36 in. [6.0 cm]) picturing the face of a subject (target) was presented to participants. The picture was introduced by a brief description of the job of the person pictured (“He/She is a Doctor/Operator working in a Hospital/Call centre”). Participants randomly received one of the eight experimental conditions: (a) picture of a very attractive woman with high status job; (b) picture of an unattractive woman with high status job; (c) picture of a very attractive woman with low status job; (d) picture of an unattractive woman with low status job; (e) picture of a very attractive man with high status job; (f) picture of an unattractive man with high status job; (g) picture of a very attractive man with low status job; (h) picture of an unattractive man with low status job.

After viewing the photograph, participants were asked to evaluate Big Five traits of target.

### Independent Variables Manipulations

#### Attractiveness

A pretest of 66 university students (33 males and 33 female; average age 23.20; SD = 3.43) received a set of 10 photographs reproducing women’s faces and ten photographs reproducing men’s faces. Participants rated the physical attractiveness of the faces on a 10-point scale. On the basis of these evaluations we chose the most attractive females (*M* = 7.64 and *M =* 7.42) and males (*M* = 7.35 and *M =* 7.34) (High Attractiveness conditions) and the less attractive ones (Females: *M* = 3.97 and *M =* 4.50; Males: *M* = 3.74 and *M =* 3.79) (Low Attractiveness conditions) as stimuli for the experiment.

#### Status

During the pretest, participants were also asked to rate a list of 14 jobs on a 5-point scale ranging from 1 (very low status) to 5 (very high status). We chose the jobs that in a previous study on a similar population have been evaluated neither typically masculine nor typically feminine (Rollero & Tartaglia, 2013). We used these evaluations to select the two jobs rated lowest (Call centre operator, *M* = 1.85) and highest (Doctor, *M* = 4.50) in status. We manipulated the status of the target in different conditions presenting the person in the photograph as a Doctor (High-status conditions) or as a Call centre operator (Low-status conditions).

### Dependent Measures

Participants were asked to evaluate the Big Five personality traits of the target by means of five single items using a Bipolar Response Scale (Woods & Hampson, 2005). Each item is made of two opposing descriptions representing the poles of a Big Five factor. A nine-point scale was placed between the two descriptions. In its original version, this scale was used to assess self-evaluations asking participants to indicate the extent to which one pole or the other best described them. We modified the instructions asking participants to evaluate the person in the photograph.

## Results

Table 1 reports the descriptive statistics of the dependent variables. We performed two-way between-group ANOVAs to determine the presence of significant effects of target’s attractiveness, status, and gender on each perceived Big Five factor. We also controlled for the effect of the respondents’ gender.

**Table 1:** Perceived Big Five Personality Traits: Descriptive Statistics

Personality Trait	*M*	*SD*	Correlations			
			1	2	3	4
1. Extraversion	4.60	2.51				
2. Agreeableness	4.27	2.23	-.19**			
3. Neuroticism	5.68	2.19	-.35**	.06		
4. Conscientiousness	5.26	2.40	-.01	-.13**	-.01	
5. Openness	3.96	2.10	.02	.16**	.01	-.16**

**p < .01.

### Extraversion

**Figure 1:**
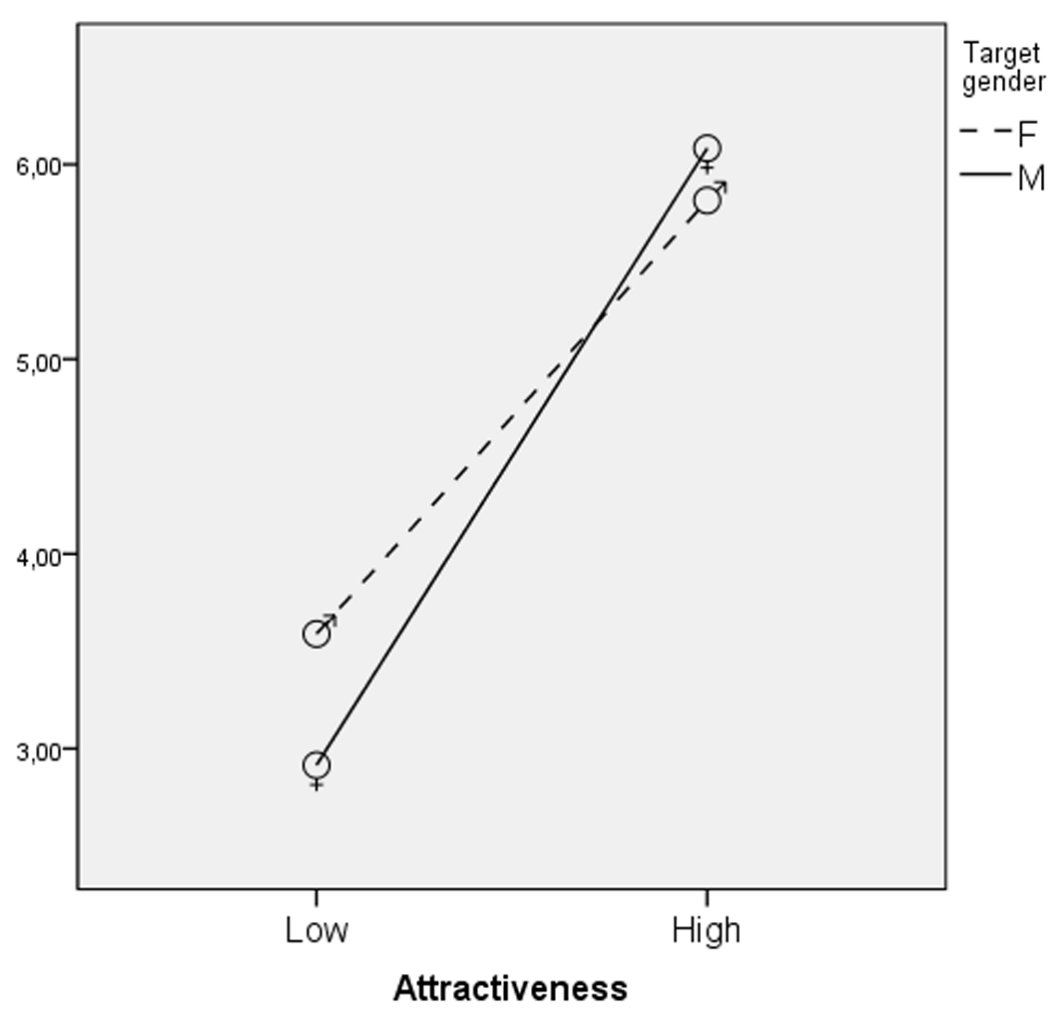
Interaction of attractiveness and target gender: Evaluation of the target in terms of extraversion mean scores.

As predicted by Hypothesis 1, attractiveness increased the perception of Extraversion, *F*(1,451) = 187.68, *p* < .01, eta square = .29. The attractive targets (*M* = 5.96; *SD* = 2.26) were considered more extraverted than the unattractive ones (*M* = 3.24; *SD* = 1.96). Contrary to what was expected, target status did not have any effect. An interaction effect between attractiveness and target gender was found, *F*(1,451) = 5.73, *p* < .02, eta square = .01 (see Figure 1 and Table 2) showing that the effect of attractiveness was stronger for male targets.

**Table 2:** Perceived extraversion: Simple-effects comparisons for the interaction of attractiveness and target gender.

Target	*M*	*SD*	Post Hoc (LSD)
A. Unattractive male targets	2.92	1.79	A-C *p* < .01
			A-D *p* < .01
B. Unattractive female targets	3.56	2.07	B-C *p* < .01
			B-D *p* < .01
C. Attractive male targets	6.08	2.26	C-A *p* < .01
			C-B *p* < .01
D. Attractive female targets	5.83	2.26	D-A *p* < .01
			D-B *p* < .01

### Agreeableness

As predicted by Hypothesis 2, target status decreased the perception of Agreeableness, *F*(1,451) = 10.81, *p* < .01, eta square = .02. The low-status targets (*M* = 4.59; *SD* = 2.25) were considered more agreeable than the high-status targets (*M* = 3.94; *SD* = 2.15). No influence of attractiveness was found. Concerning target gender, male targets (*M* = 4.74; *SD* = 2.23) were judged more agreeable than females (*M* = 3.77; *SD* = 2.11), *F*(1,451) = 22.33, *p* < .01, eta square = .05. No interaction effect was found.

### Neuroticism

As predicted by Hypothesis 3, attractiveness decreased the perception of Neuroticism, *F*(1,451) = 52.72, *p* < .01, eta square = .10. The attractive targets (*M* = 4.99; *SD* = 2.03) were considered less neurotic than the unattractive ones (*M* = 6.37; *SD* = 2.14). Contrary to what was predicted, target status did not have any effect. As expected, female targets (*M* = 6.14; *SD* = 1.94) were judged more neurotic than males (*M* = 5.21; *SD* = 2.33), *F*(1,451) = 23.98, *p* < .01, eta square = .05. No interaction effect was found.

### Conscientiousness

As expected status and target gender influenced the perception of Conscientiousness. The high-status targets (*M* = 5.69; *SD* = 2.41) were considered more conscientious than the low-status targets (*M* = 4.83; *SD* = 2.32), *F*(1,451) = 16.72, *p* < .01, eta square = .04. Female targets (*M* = 5.59; *SD* = 2.32) were evaluated more conscientious than the male targets (*M* = 4.94; *SD* = 2.44), *F*(1,451) = 9.43, *p* < .01, eta square = .02.

**Figure 2:**
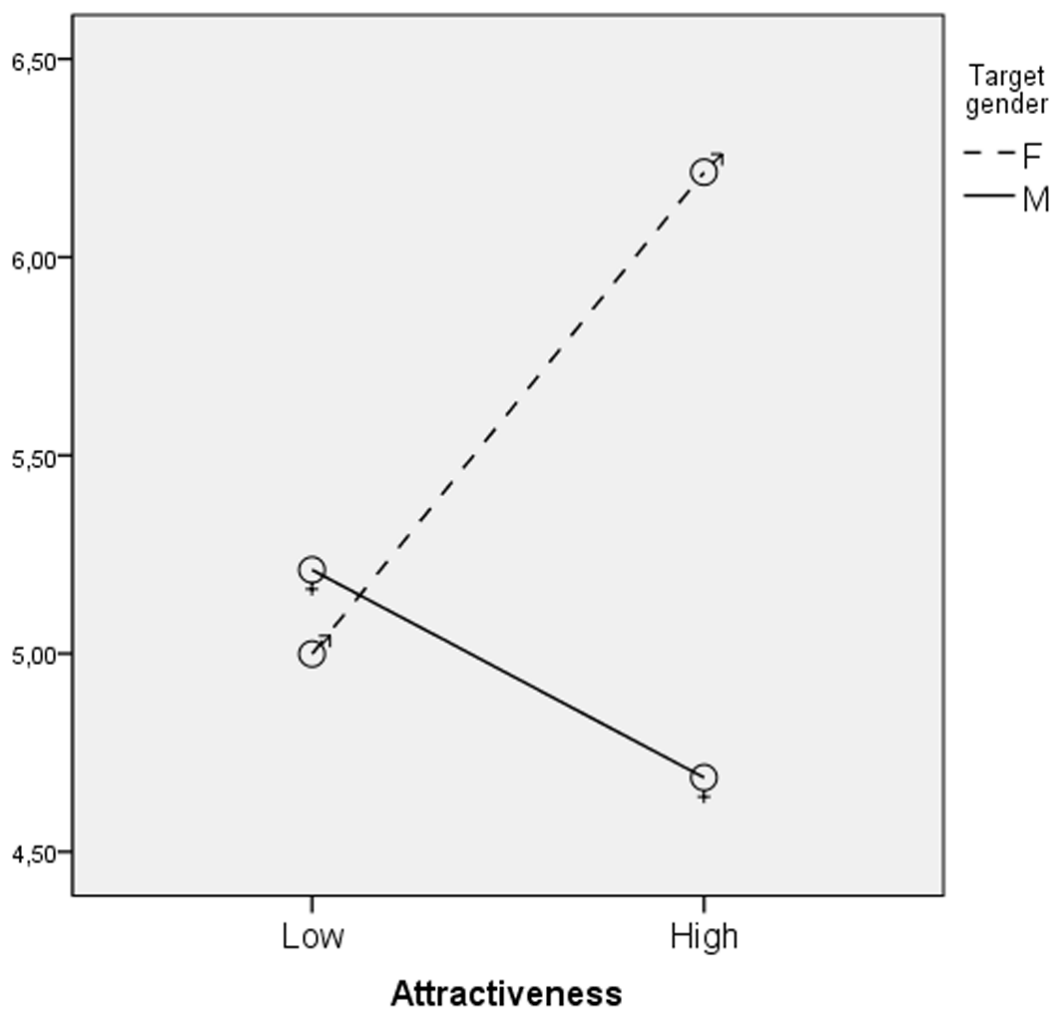
Interaction of attractiveness and target gender: Evaluation of the target in terms of conscientiousness mean scores.

**Figure 3:**
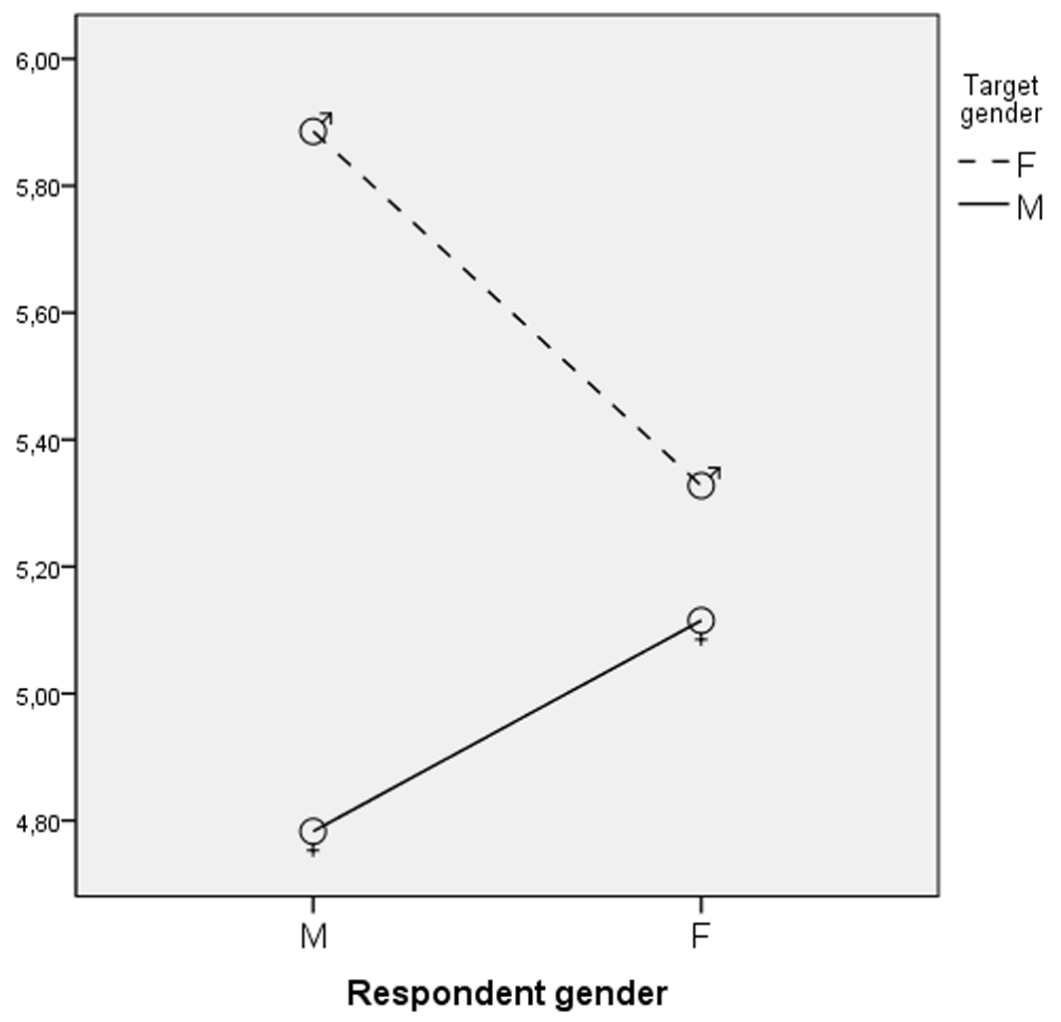
Interaction of respondent gender and target gender: Evaluation of the target in terms of conscientiousness mean scores.

An interaction effect between attractiveness and target gender was found, *F*(1,451) = 16.50, *p* < .01, eta square = .04 (see Figure 2). The hypothesized positive effect of attractiveness was confirmed only for female targets. Attractive women were considered more conscientious than all the other targets (see Table 3). Another interaction effect between gender of participants and target gender was found, *F*(1,451) = 4.32, *p* < .05, eta square = .01 (see Figure 3). Male participants evaluated women more conscientious than men (see Table 4).

**Table 3:** Perceived Conscientiousness: Simple-Effects Comparisons for the Interaction of Attractiveness and Target Gender.

Target	*M*	*SD*	Post Hoc (LSD)
A. Unattractive male targets	5.20	2.56	A-D *p* < .01
B. Unattractive female targets	4.96	2.41	B-D *p* < .01
C. Attractive male targets	4.70	2.30	C-D *p* < .01
D. Attractive female targets	6.20	2.06	D-A *p* < .01
			D-B *p* < .01
			D-C *p* < .01

**Table 4:** Perceived Conscientiousness: Simple-Effects Comparisons for the Interaction of Respondent Gender and Target Gender.

Respondent Gender and Target Gender	*M*	*SD*	Post Hoc (LSD)
A. Male respondents evaluating male targets	4.79	2.30	A-B *p* < .01
B. Male respondents evaluating female targets	5.90	2.28	B-A *p* < .01
C. Female respondents evaluating male targets	5.10	2.58	
D. Female respondents evaluating female targets	5.33	2.33	

### Openness

**Figure 4:**
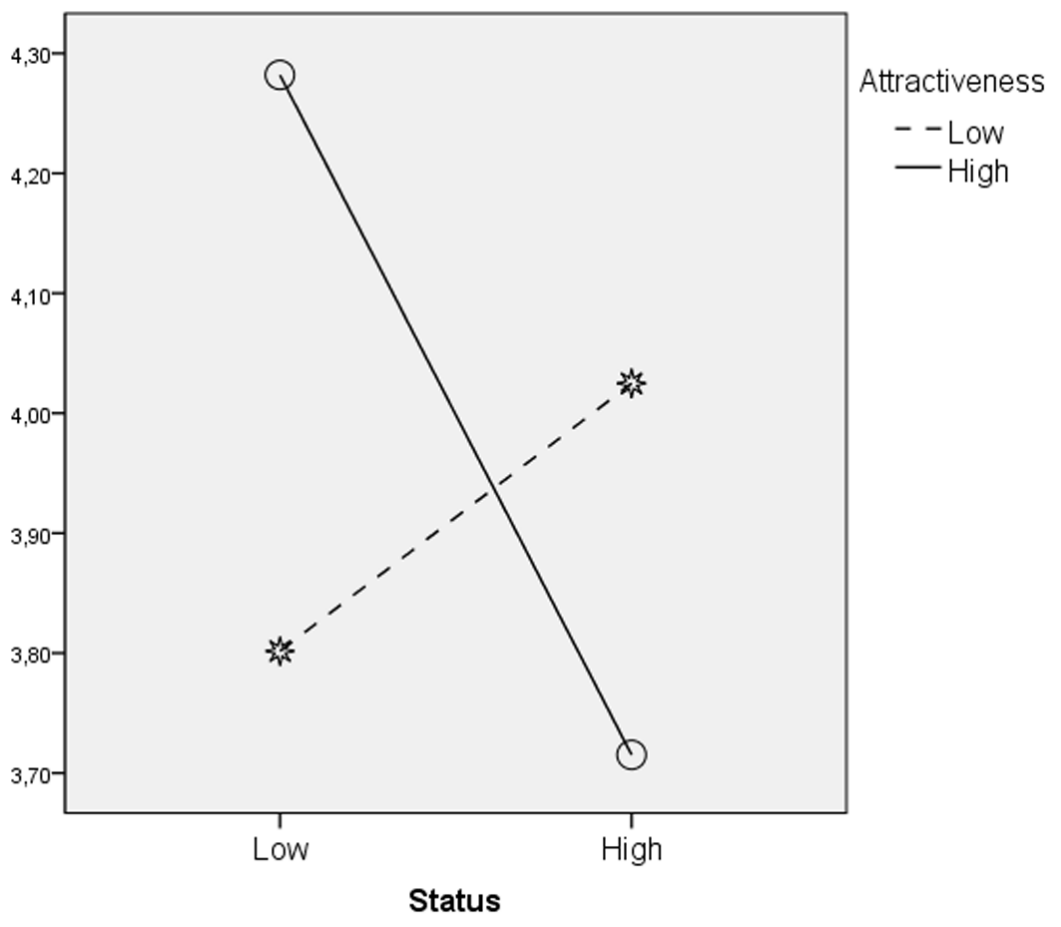
Interaction of status and attractiveness: Evaluation of the target in terms of openness mean scores.

**Figure 5:**
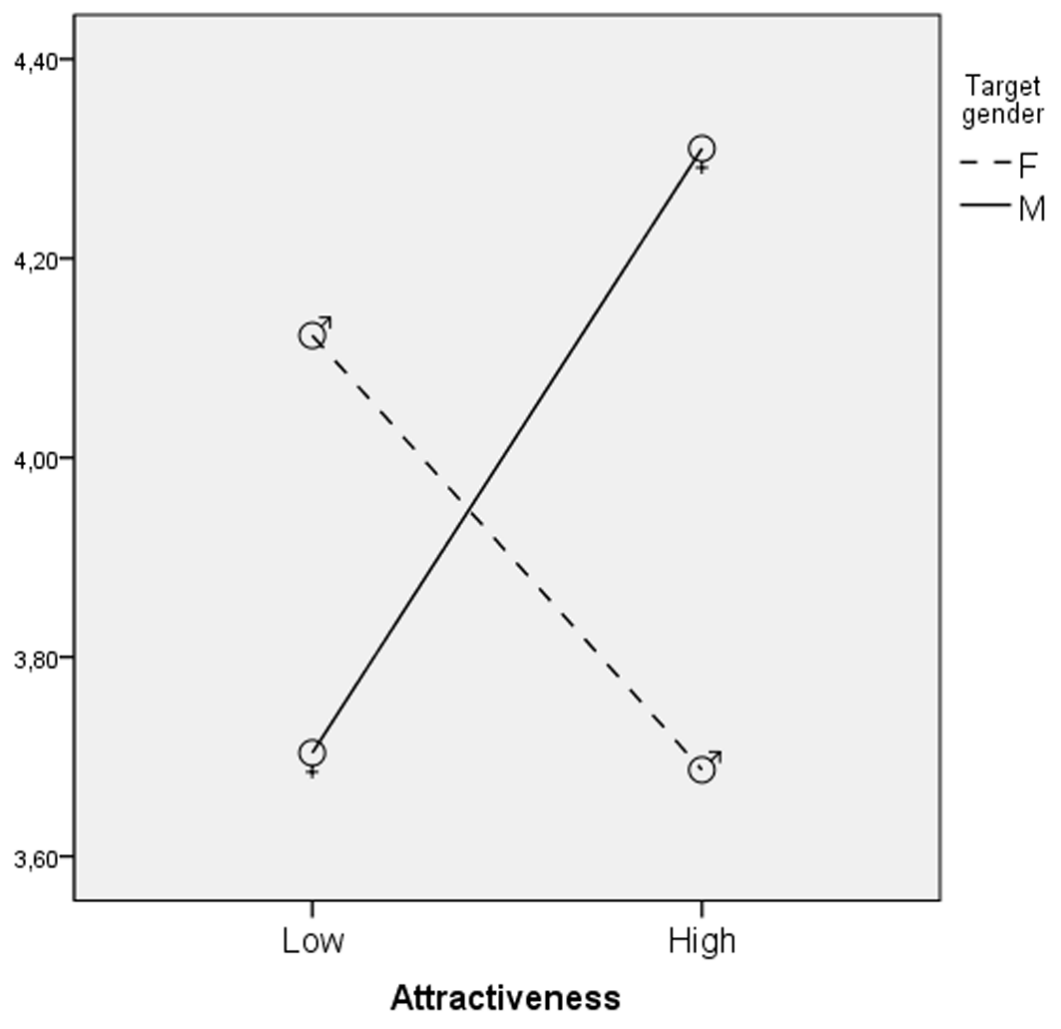
Interaction of attractiveness and target gender: Evaluation of the target in terms of openness mean scores.

There were no main effects of independent variables on perceived Openness. The hypothesized effect of attractiveness was in interaction with target status, *F*(1,451) = 4.11, *p* < .05, eta square = .01, and with target gender, *F*(1,451) = 7.17, *p* < .01, eta square = .02 (see Figures 4 and 5). Low-status attractive targets were considered more open than high-status attractive targets (see Table 5). Attractive male targets were evaluated more open than unattractive male targets and attractive female targets (see Table 6). Attractiveness seemed to be a benefit for low-status and male targets.

**Table 5:** Perceived Openness: Simple-Effects Comparisons for the Interaction of Status and Attractiveness.

Target	*M*	*SD*	Post Hoc (LSD)
A. Low-status unattractive targets	3.78	2.03	
B. Low-status attractive targets	4.29	2.11	B-D *p* < .05
C. High-status unattractive targets	4.03	2.13	
D. High-status attractive targets	3.72	2.12	D-B *p* < .05

**Table 6:** Perceived Openness: Simple-Effects Comparisons for the Interaction of Attractiveness and Target Gender.

Target	*M*	*SD*	Post Hoc (LSD)
A. Unattractive male targets	3.71	2.16	A-C *p* < .05
B. Unattractive female targets	4.11	1.98	
C. Attractive male targets	4.31	2.10	C-A *p* < .05
			C-D *p* < .05
D. Attractive female targets	3.69	2.12	D-C *p* < .05

## Discussion

The present study examined stereotypic processes that foster inferences on others’ personality. Specifically, we considered whether attractiveness, occupational status and gender of the target affect his/her personality evaluation. Results revealed that judgments of all Big Five personality dimensions are affected by these variables.

In general, results are in line with the ‘beauty is good’ effect (Dion et al., 1972), as people seem to believe that physical attractiveness implies positive personality traits, but the effects of attractiveness are different for men and women. Consistently with previous studies (Ćurković & Franc, 2010; Langlois et al., 2000), the attractive targets were judged as extraverted and emotionally stable. Different interactive effects of targets' physical attractiveness and gender were confirmed on the judgments of Extraversion, Conscientiousness and Openness. For Extraversion the effect of attractiveness is the same for women and men but is stronger for male targets. Attractiveness has a positive effect on Conscientiousness only for women whereas it increases Openness only for men.

Thus, overall the “beauty is good effect” seems to be greater for men. We can interpret this finding thinking that in contemporary western societies the female attractiveness easily implies the sexualisation of women (Liss, Erchull, & Ramsey, 2011; Nowatzki & Morry, 2009; Rollero, 2013). Indeed, while physical attractiveness has been shown to generate a broadly favorable impression of both men and women, investigations of the traits associated with women’s sexiness suggest a stereotype that is damaging for other aspects: for example, it is poor match for high-status jobs (Glick, Larsen, Johnson, & Branstiter 2005; Rollero & Tartaglia, 2013; Tartaglia & Rollero, 2015). The job of our targets was salient, as targets of our study were described only in relation to their profession. For women in this domain attractiveness could be partly damaging. Actually our results showed an interactive effect of target status and attractiveness on Openness judgment indicating that being attractive increases that positive trait only for low-status targets. This result is consistent with the “beauty is beastly effect” affirming that attractiveness is damaging for women applying for masculine jobs for which physical appearance is perceived to be unimportant (Johnson, Podratz, Dipboye, & Gibbons, 2010), usually the high–status jobs.

The influence of occupational status is more controversial. Although literature was confirmed for Agreeableness (lower for high-status targets) and Conscientiousness (higher for high-status targets), status had no influence on Extraversion and Neuroticism. This may be due to the specific inference task in which participants were involved. Existing literature is based on the Big Five dimensions as predictive elements of status attainment in the occupational domain, whereas in the present study people were asked to deduce personality traits from the occupational status. Furthermore, the chosen low status job (call centre operator) is an occupation requiring communicative competences and relational attitudes that are strictly linked to Extraversion. This fact might explain why in present case high status targets were not considered more extraverted than their low status counterparts were.

From a gender perspective, the present study confirms stereotypes regarding women as more neurotic and more conscientious (Costa et al., 2001; McCrae & Terracciano, 2005). We obtained unexpected results concerning Agreeableness. Previous research carried out in Italy showed no gender differences on this trait (McCrae & Terracciano, 2005), whereas in our study men were considered more agreeable. Further research should address this point, as the experimental design used in this study may have fostered a specific evaluation process, matching inferences based on gender, job status, and attractiveness. Finally, the stereotypes about gender personality seem to be largely shared among men and women. Indeed, the gender of the respondents did not have any direct effect on the traits and had just one interaction effect with target gender, as male participants judged women more conscientious than men, whereas females did not. However, findings concerning the effect of respondents’ gender are explorative, as in the present study we did not focus on participants’ gender, but only controlled for its effect.

Since, to our knowledge, this was the first study that examined the combined effects of attractiveness, occupational status, and gender on the evaluation of others’ personality, further research is necessary to strengthen present findings. For example, it should be noteworthy taking into account certain traits of respondents, such as their own attractiveness, their chance for professional success, as well as their endorsement of sexist attitudes. Moreover, other dependent variables may be considered, such as evaluation of life satisfaction and well-being of targets with different characteristics. This could contribute to shed light on mechanisms of forming judgments about personality.

In sum, the present findings shed light on relevant elements that may operate in evaluation processes. As the social expectations perspective has argued (see Langlois et al., 2000), social stereotypes affect people’s specific expectations about others. Facial appearance, as well as status, elicits social stereotypes that in turn create expectations for the characteristics of the target.
